# The Comparison of Different Constitutive Laws and Fiber Architectures for the Aortic Valve on Fluid–Structure Interaction Simulation

**DOI:** 10.3389/fphys.2021.682893

**Published:** 2021-06-24

**Authors:** Li Cai, Ruihang Zhang, Yiqiang Li, Guangyu Zhu, Xingshuang Ma, Yongheng Wang, Xiaoyu Luo, Hao Gao

**Affiliations:** ^1^NPU-UoG International Cooperative Lab for Computation and Application in Cardiology, Northwestern Polytechnical University, Xi'an, China; ^2^Xi'an Key Laboratory of Scientific Computation and Applied Statistics, Xi'an, China; ^3^School of Energy and Power Engineering, Xi'an Jiaotong University, Xi'an, China; ^4^College of Bioengineering, Chongqing University, Chongqing, China; ^5^School of Mathematics and Statistics, University of Glasgow, Glasgow, United Kingdom

**Keywords:** aortic valve, hybrid immersed boundary/finite element method, fluid-structure interaction, mechanical properties, dynamic behaviors, hemodynamic performance, the constitutive law

## Abstract

Built on the hybrid immersed boundary/finite element (IB/FE) method, fluid–structure interaction (FSI) simulations of aortic valve (AV) dynamics are performed with three different constitutive laws and two different fiber architectures for the AV leaflets. An idealized AV model is used and mounted in a straight tube, and a three-element Windkessel model is further attached to the aorta. After obtaining *ex vivo* biaxial tensile testing of porcine AV leaflets, we first determine the constitutive parameters of the selected three constitutive laws by matching the analytical stretch–stress relations derived from constitutive laws to the experimentally measured data. Both the average error and relevant R-squared value reveal that the anisotropic non-linear constitutive law with exponential terms for both the fiber and cross-fiber directions could be more suitable for characterizing the mechanical behaviors of the AV leaflets. We then thoroughly compare the simulation results from both structural mechanics and hemodynamics. Compared to the other two constitutive laws, the anisotropic non-linear constitutive law with exponential terms for both the fiber and cross-fiber directions shows the larger leaflet displacements at the opened state, the largest forward jet flow, the smaller regurgitant flow. We further analyze hemodynamic parameters of the six different cases, including the regurgitant fraction, the mean transvalvular pressure gradient, the effective orifice area, and the energy loss of the left ventricle. We find that the fiber architecture with body-fitted orientation shows better dynamic behaviors in the leaflets, especially with the constitutive law using exponential terms for both the fiber and cross-fiber directions. In conclusion, both constitutive laws and fiber architectures can affect AV dynamics. Our results further suggest that the strain energy function with exponential terms for both the fiber and cross-fiber directions could be more suitable for describing the AV leaflet mechanical behaviors. Future experimental studies are needed to identify competent constitutive laws for the AV leaflets and their associated fiber orientations with controlled experiments. Although limitations exist in the present AV model, our results provide important information for selecting appropriate constitutive laws and fiber architectures when modeling AV dynamics.

## 1. Introduction

There are up to 250,000 heart valves repaired and replaced each year worldwide (Yoganathan et al., 2004). Among these, aortic valve (AV) diseases have become the second-leading cause of cardiovascular diseases due to their high morbidity and mortality (Go et al., 2013). Major AV diseases include aortic stenosis, calcification, regurgitation, etc. (Franzone et al., 2016). Current treatments mainly focus on surgical repair and valve replacement. However, difficulties and risks exist in surgical procedures. Numerical simulations of the AV dynamics can assess the hemodynamic performance, predict the effectiveness, and persistence of surgical treatments, thereby help AV disease management (Mohammadi et al., 2016; Chen and Luo, 2018).

The AV locates at the root of the supporting aorta and provides a path of oxygenated blood to be pumped from the heart into the systemic circulation while preventing blood from flowing back from the aorta into the left ventricle (LV). The AV is composed of three relatively equal-sized semi-lunar leaflets, whose attachment forms the valve annulus and three bulges comprising the aortic sinuses. The dynamics of AV are driven by the pressure gradient between the LV and the aorta (Mohammadi et al., 2016). For example, in the systolic phase, the pressure of the LV is higher than that of the aorta, resulting in the AV opening and blood flowing from the LV into the aorta. In diastole, the AV closes as the LV pressure decreases.

Early numerical studies of the AV mainly focused on structural analysis using the finite element method (FEM). Since the mid-1970s, researchers have begun to simulate the AV based on simple geometries. In 1973, Gould et al. (1973) constructed three different geometries for the closed AV leaflets and concluded that changes in the leaflets geometry lead to great changes in the stress field. After that, Chong et al. (1978) studied the stress state of porcine AV leaflets using FEM. Since the 1990s', researchers have begun to use commercial software to analyze AV behaviors and stress distributions (Black et al., 1991). Kunzelman et al. developed the first three-dimensional FE model of the mitral valve (MV) and analyzed the deformation and stress patterns of the MV using LS-DYNA (Livermore Software Technology Corporation, Livermore, CA), followed by a series of studies, they have provided a deep understanding of normal and abnormal MV anatomy and function (Kunzelman et al., 1993, 1997, 2007). Meanwhile, their numerical MV model was the first to use patient-specific magnetic resonance imaging (MRI) data rather than idealized geometry.

Because of the strong interaction between heart valves and blood flow, fluid–structure interaction (FSI) methods were introduced. Chew et al. (1999) developed a three-dimensional (3D) model of a bioprosthetic porcine valve with non-linear material properties, and the FSI simulation was implemented through the Arbitrary Lagrangian-Eulerian (ALE) method. De Hart et al. (2003a,b) developed a 3D model of the AV using the fictitious domain (FD) method. Van Loon et al. (2004) used a combined ALE and FD method to validate the FSI models in simulating the healthy and stenotic AV. Besides, Weinberg et al. (2010) established a multiscale FSI model of the AV to capture the mechanical behaviors of the AV. Morganti et al. (2015) utilized a patient-specific valve geometry model to simulate the closure of the AV by isogeometric analysis. Mohammadi et al. (2016) reviewed the numerical methods for studying the hemodynamics of the AV, especially the FSI method.

Due to the large deformation of the valve leaflets, severe distortion, and deterioration may exist in the fluid mesh, and the ALE method can be challenging because of the frequent mesh regeneration (Gao et al., 2017b). To overcome such difficulty, the immersed boundary (IB) method was introduced by Peskin to simulate heart valve dynamics (Peskin, 2002). The IB method greatly simplifies the mesh regeneration and facilitates the numerical simulation of large deformation in the elastic structure. Griffith et al. (2009) applied the IB method to simulate the fluid dynamics of heart valves, including a natural AV and a chorded prosthetic MV. After that, Griffith (2012) used a staggered-grid version of the IB method to simulate the AV dynamics over multiple cardiac cycles. Ma et al. (2013) utilized this IB method to perform the FSI simulation for a human anatomical MV model obtained from *in vivo* MRI data. The immersed finite element (IFE) method was an extension of the IB method, where the FE approximations were applied to the Eulerian and Lagrangian equations. Built on the classical IB method, Griffith and Luo (2017) discretized the immersed structure using the FE method and the incompressible Navier–Stokes equation using the finite difference method, which is the hybrid finite difference/finite element immersed boundary (IB/FE) method. By using the IB/FE method, Gao et al. (2014) simulated the dynamic behaviors of a human MV reconstructed from *in vivo* MRI data, then extended to a coupled MV–LV model (Gao et al., 2017a). Feng et al. (2019) achieved the FSI simulation of a coupled left atrium—MV model by the IB/FE framework. The same IB/FE framework has also been applied to AV modeling. For example, Flamini et al. (2016) studied the effects of the aortic root on the AV dynamics, and their results showed reasonable agreement with the physiological measurements. Hasan et al. (2017) constructed a realistic, three-dimensional anatomical IB/FE model of the aortic root and ascending aorta. Recently, Lee et al. (2020a) performed FSI simulations of the porcine AV and the bovine pericardial valve, with the computational results being in excellent agreement with the experimental data. Lee et al. (2020b) further studied the experimental and IB/FE model of bioprosthetic aortic valves (BAVs).

It has been widely acknowledged that the material properties of heart valves can play an important role in valvular function. Heart valve tissue mainly consists of collagen and elastin, which is usually considered to be a material of anisotropy, hyperelasticity, non-linearity, and incompressibility (Weinberg and Kaazempur-Mofrad, 2005). To characterize the mechanical properties of the valve leaflets, the formulation of the constitutive law of heart valve tissue should be determined according to its underlying biological structure. Martin and Sun (2012) studied the biomechanical properties of the AV leaflets of the human, porcine, and ovine, and they found that the aged human AV leaflets were stiffer than the porcine and ovine AV leaflets. Pham et al. (2017) used the planar biaxial testing to characterize the mechanical and structural properties of four different heart valves, and found that great differences exist in thickness, stiffness, and anisotropy. Wang et al. (2012, 2015) adopted the anisotropic hyperelastic material model to describe the mechanical properties of the AV tissues, including the leaflets, the sinus, the ascending aorta, and the myocardium. Mao et al. (2016) compared an anisotropic and an isotropic leaflet material model in transcatheter AV simulations, and their results suggested that the isotropic model showed a stiffer leaflet behavior in the radial direction than the anisotropic model. Recent reviews of material properties of valvular tissues can be found in Sun et al. (2014).

Furthermore, the fiber architecture of the valve leaflets plays an essential role in the mechanical function of the AV. Early studies for the fiber orientation distribution of the human heart relied on the projections and the least–square fitting methods. For example, Toussaint et al. (2010) reconstructed the complete 3D human cardiac fiber architecture using a curvilinear interpolation of diffusion tensor images. At present, two different approaches are mainly used for reconstructing the fiber distribution in soft tissue. The first method is the rule-based method by assuming that collagen fibers align circumferentially based on a cylindrical coordinate system (Gao et al., 2014; Hasan et al., 2017). Rule-based methods have been widely used in soft tissue modeling, including arteries (Qi et al., 2015) and heart (Wang et al., 2013; Gao et al., 2015; Guan et al., 2020). The other approach is to map fiber distributions from *in/ex vivo* experimental measurements. For example, Aggarwal et al. (2013) used a spline-based method to obtain the fiber structure by mapping them from histological analysis of AV specimen.

There is a lack of comparative study of constitutive laws of AV in FSI simulations. Our previous studies on MV suggested that different constitutive laws can affect MV dynamics (Cai et al., 2019). Thus, in this study, we analyze the effects of three different constitutive laws and two different fiber architectures on AV dynamics and hemodynamics. We first construct an idealized AV model mounted in a straight tube coupled with a three-element Windkessel model for systemic circulation. To characterize the material properties of the leaflets, we first measured the stiffness of porcine AV samples using biaxial tensile testing, then three different constitutive laws are considered from published studies. We then simulate the AV dynamics using the IB/FE method and finally analyze the leaflets dynamics and hemodynamic performance in six different cases.

## 2. Methods

### 2.1. The AV Model

#### 2.1.1. The Computational Model

**Figure 1:**
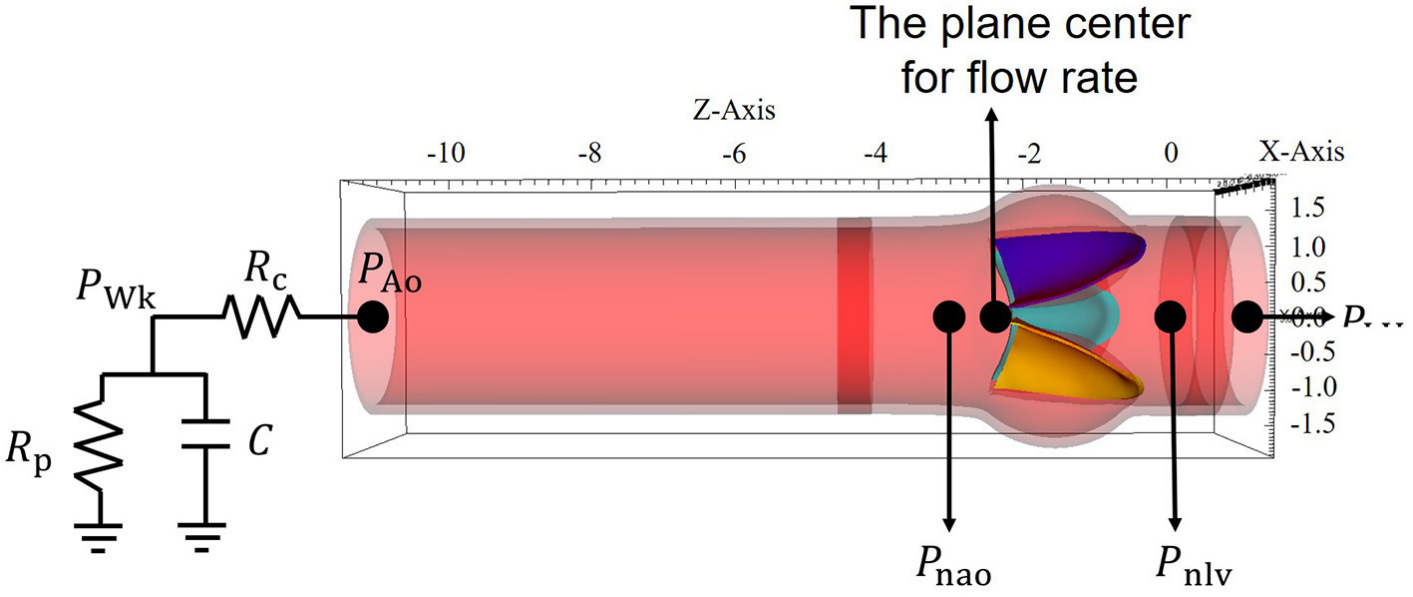
The geometric model of the aortic valve (AV).

Figure 1 shows the AV model mounted in a straight tube. This idealized AV model is constructed according to the porcine pericardial valve with a leaflet thickness of 0.04 cm (Zhu et al., 2017). The straight tube has a total length of 13 cm, with the inner radius 1.3 cm, and the wall thickness 0.15 cm, which is also similar to the AV model in Flamini et al. (2016). Besides, based on a novel expanded-polytetrafluoroethylene (ePTFE) stentless tri-leaflet valve, Zhu et al. (2017) experimentally assessed the dynamic and hemodynamic performance of the AV, which provides the reference values for validating this AV model.

In the previous work, Griffith (2012) used a three-element Windkessel model for providing dynamic pressure loading. Here, we follow the same approach as shown in Figure 1. The three-element Windkessel model consists of the characteristic resistance *R*_c_, the peripheral resistance *R*_p_, and the arterial compliance *C*. Let *P*_LV_ denote the left ventricular pressure (inlet pressure), and let *P*_Ao_ denote the aortic pressure (outlet pressure) obtained from the three-element Windkessel model. *P*_Wk_ is the pressure stored in the Windkessel model. Assuming *Q*_Ao_ is the flow rate through the outlet boundary, according to the relationship between the pressure, the flow rate, and the resistance, we have (Griffith, 2012).

(1) $$\begin{array}{r} C\frac{\text{d}P_{\text{Wk}}}{\text{d}t}+\frac{P_{\text{Wk}}}{R_{\text{p}}}=Q_{\text{Ao}}, \end{array}$$

(2) $$\begin{array}{r} P_{\text{Ao}}=Q_{\text{Ao}}R_{\text{c}}+P_{\text{Wk}}. \end{array}$$

The details of numerical implementation of this Windkessel model can be found in Griffith (2012). In the following simulation, we set $R_{\text{c}}=0.033\text{ mmHg m}{\text{l}}^{-1}\text{s}$, $R_{\text{p}}=0.79\text{ mmHg m}{\text{l}}^{-1}\text{s}$, *C* = 1.75 ml mmHg^−1^, and the initial pressure *P*_Wk_ = 85 mmHg and *P*_Ao_ = *P*_Wk_, which correspond to the human “Type A” beat in the work of Stergiopulos et al. (1999).

#### 2.1.2. The Fiber Architectures of AV Leaflets

**Figure 2:**
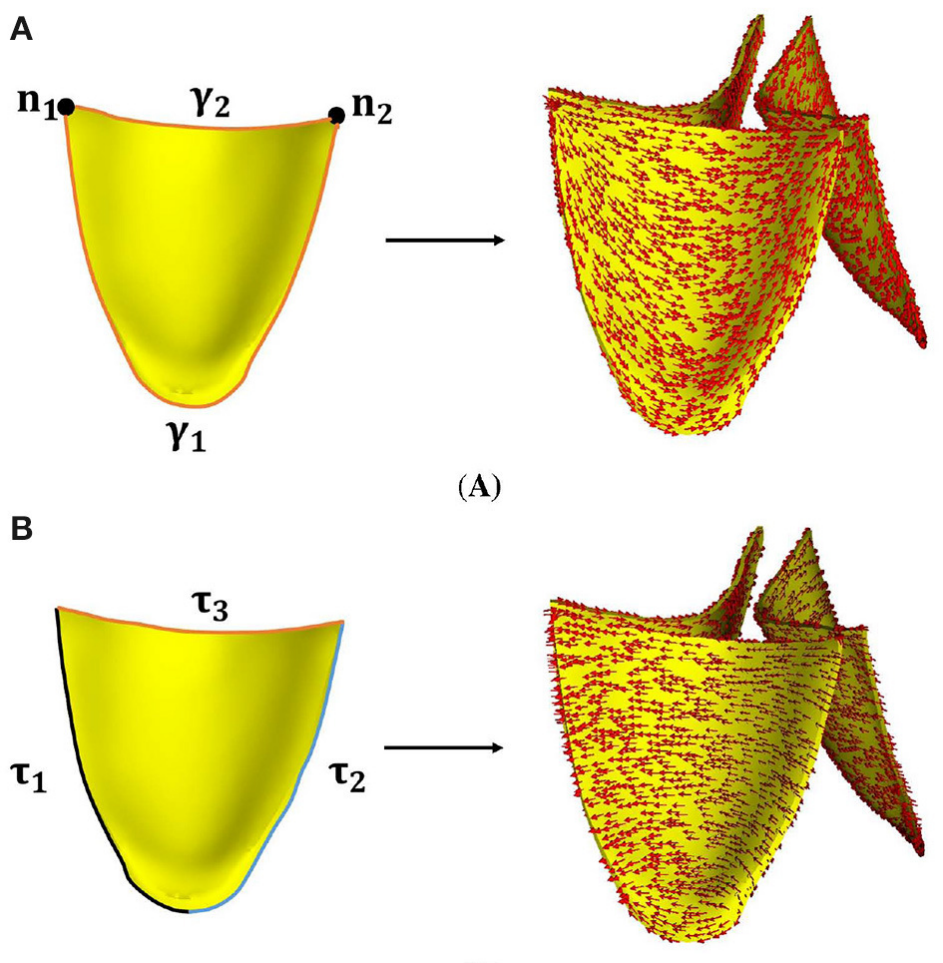
Two different fiber architectures of AV leaflets, **(A)** FD1, **(B)** FD2.

Because of lacking experimental data on collagen orientations in the leaflets, the rule-based method is used to construct two different fiber architectures in AV leaflets, which are further denoted as FD1 and FD2 as shown in Figure 2. Both fiber architectures are circumferentially aligned in general and constructed by solving a Poisson-type system of a scalar field *u* (Wong and Kuhl, 2014; Guan et al., 2020). FD1 is body-fitted, and FD2 is simply the circumferential direction. In detail, the Poisson system for FD1 is defined as

(3) $$\begin{array}{l} \text{FD1}:\{\begin{array}{l} \nabla^{2}u=0,\text{in }B,\\ u{|}_{n_{1}}=1,\\ u{|}_{n_{2}}=0,\\ \frac{\partial u}{\partial n}{|}_{\Gamma^{N}}=0, \end{array} \end{array}$$

in which B represents the leaflet, *n*_1_ and *n*_2_ are the two corner points indicated in Figure 2A, and Γ^*N*^ is the surface of the leaflet. The collagen fiber direction is defined as f = ∇*u*/|∇*u*|.

To construct FD2, the Poisson system is defined as

(4) $$\begin{array}{l} \text{FD2}:\{\begin{array}{l} \nabla^{2}u=0,\text{in }B,\\ u{|}_{\tau_{1}}=0,\\ u{|}_{\tau_{2}}=1,\\ \frac{\partial u}{\partial n}{|}_{\Gamma^{N}}=0, \end{array} \end{array}$$

in which τ_1_ and τ_2_ are the two surfaces connecting the leaflet to the aorta, and symmetric about the central line of the leaflet as shown in Figure 2B, Γ^*N*^ represents the remaining surfaces of the leaflet. The corresponding fiber direction is also given by ∇*u*/|∇*u*|.

From Figure 2, it can be seen that the fiber architecture FD1 has a body-fitted fiber orientation, which is similar to the fiber structures in Aggarwal et al. (2013); Hasan et al. (2017). The fiber architecture FD2 is simply the circumferential direction, which has been used by Fan et al. (2013).

### 2.2. The IB/FE Method

The IB/FE method (Gao et al., 2014; Griffith and Luo, 2017) is employed here to simulate the AV dynamics, which uses the FE discretization for the immersed structure and the finite difference discretization for the viscous incompressible fluid. Let **X** = (*X*_1_, *X*_2_, *X*_3_) ∈ *U* denote Lagrangian material coordinates, and let **x** = (*x*_1_, *x*_2_, *x*_3_) ∈ Ω represent physical coordinates, in which *U* ⊂ *R*^3^ means the Lagrangian coordinate domain, and Ω ⊂ *R*^3^ denotes the fixed physical domain of the FSI system. Let **χ**(**X**, *t*) ∈ Ω denote the physical position of structure point **X** at time *t*, then **χ**(*U, t*) ⊂ Ω is the physical domain of the structure at time *t*, whereas the physical domain occupied by the fluid at time *t* is Ω − **χ**(*U, t*). The governing equations of the FSI system are given as

(5) $$\begin{array}{l} \rho \left(\frac{\partial \text{u}}{\partial t}\left(\text{x},t\right)+\text{u}\left(\text{x},t\right)\cdot \nabla \text{u}\left(\text{x},t\right)\right)=-\nabla p\left(\text{x},t\right)+\mu \nabla^{2}\text{u}\left(\text{x},t\right)\\ \;+{\text{f}}^{\text{e}}\left(\text{x},t\right), \end{array}$$

(6) $$\begin{array}{l} \nabla \cdot \text{u}\left(\text{x},t\right)=0, \end{array}$$

(7) $$\begin{array}{l} \frac{\partial \chi }{\partial t}\left(\text{X},t\right)=\int_{\Omega }\text{u}\left(\text{x},t\right)\delta \left(\text{x}-\chi \left(\text{X},t\right)\right)\text{d}\text{x}, \end{array}$$

(8) $$\begin{array}{l} {\text{f}}^{\text{e}}\left(\text{x},t\right)=\int_{U}\nabla \cdot {\mathbb{P}}^{\text{e}}\left(\text{X},t\right)\delta \left(\text{x}-\chi \left(\text{X},t\right)\right)\text{d}\text{X}\\ \;-\int_{\partial U}{\mathbb{P}}^{\text{e}}\left(\text{X},t\right)\text{N}\left(\text{X}\right)\delta \left(\text{x}-\chi \left(\text{X},t\right)\right)\text{d}A\left(\text{X}\right), \end{array}$$

where **u**(**x**, *t*) is the Eulerian velocity field, *p*(**x**, *t*) is the Eulerian pressure field, ρ = 1.0 g/ml is the mass density, μ = 4cP is the fluid dynamic viscosity, **f**^e^(**x**, *t*) is the Eulerian elastic force density, and δ(**x**) = δ(*x*_1_)δ(*x*_2_)δ(*x*_3_) is the three-dimensional Dirac delta function. ${\mathbb{P}}^{\text{e}}=\frac{\partial W}{\partial 𝔽}$ is the first Piola–Kirchhoff stress tensor, in which 𝔽 is the deformation gradient related to structural deformation. **N**(**X**) is the outer normal vector of the Lagrangian coordinate domain *U*, and d*A*(**X**) denotes the area element in the reference configuration.

The total Cauchy stress tensor of the FSI system is

(9) $$\begin{array}{l} \sigma \left(\text{x},t\right)=\sigma^{\text{f}}\left(\text{x},t\right)+\{\begin{array}{l} \sigma^{\text{e}}\left(\text{x},t\right)\text{ for }\text{x}\in \chi \left(U,t\right),\\ 0\text{ otherwise}, \end{array} \end{array}$$

in which **σ**^f^ = −*p*𝕀 + μ[∇**u** + (∇**u**)^T^] is the Cauchy stress tensor of the viscous incompressible fluid, *I* is the identity matrix, and **σ**^e^ is the elastic Cauchy stress tensor related to the first Piola–Kirchhoff stress tensor ℙ^e^, that is **σ**^e^ = *J*^−1^ℙ^e^𝔽^T^ with *J* = det(𝔽).

### 2.3. The Constitutive Laws

In this study, we consider the valvular tissue to be incompressible, anisotropic, hyperelastic (Weinberg and Kaazempur-Mofrad, 2005), and use three different constitutive laws to characterize the mechanical properties of the AV leaflets, which are denoted as W1, W2, and W3. The constitutive law W1 was used to characterize the mechanical properties of the AV tissue first by Wang et al. (2012). The constitutive law W2 was first proposed by Prot et al. (2010) for modeling the mechanical behaviors of healthy MV tissue, and the constitutive law W3 was used to model human MV leaflets first by Gao et al. (2014). The corresponding strain-energy functions are

(10) $$\begin{array}{l} \text{W1}=C_{10}\left({\text{e}}^{C_{01}\left(I_{1}-3\right)}-1\right)+\frac{k_{1}}{2k_{2}}\left[{\text{e}}^{k_{2}{\left(I_{4}-1\right)}^{2}}-1\right], \end{array}$$

(11) $$\begin{array}{l} \text{W2}=\mu \left(I_{1}-3\right)+c_{0}\left[{\text{e}}^{c_{1}{\left(I_{1}-3\right)}^{2}+c_{2}{\left(I_{4}-1\right)}^{4}}-1\right], \end{array}$$

(12) $$\begin{array}{l} \text{W3}=C_{1}\left(I_{1}-3\right)+\frac{a}{2b}\left[e^{b{\left(I_{4}-1\right)}^{2}}-1\right], \end{array}$$

where *C*_10_, *C*_01_, *k*_1_, *k*_2_ are the material parameters of Equation (10). Similarly, μ, *c*_0_, *c*_1_, *c*_2_ are the material parameters of Equation (11), and *C*_1_, *a, b* are the non-negative parameters in Equation (12). *I*_1_ = trace(ℂ) is the first strain invariant of the right Cauchy–Green deformation tensor ℂ = 𝔽^*T*^𝔽. *I*_4_ = **f**_**0**_·(ℂ**f**_**0**_) is the squared stretch along the fiber direction, with **f**_**0**_ the fiber direction in the reference state and **f** = 𝔽**f**_**0**_ the fiber direction in the current state. The corresponding Cauchy stress tensors are

(13) $$\begin{array}{l} \begin{array}{c} \sigma^{\text{W1}}=-p𝕀+2C_{10}C_{01}{\text{e}}^{C_{01}\left(I_{1}-3\right)}𝔹+2k_{1}\left(I_{4}-1\right){\text{e}}^{k_{2}{\left(I_{4}-1\right)}^{2}}\text{f}⊗\text{f}, \end{array} \end{array}$$

(14) $$\begin{array}{l} \begin{array}{c} \sigma^{\text{W2}}=-p𝕀+\left(2\mu +4c_{0}c_{1}\left(I_{1}-3\right){\text{e}}^{c_{1}{\left(I_{1}-3\right)}^{2}+c_{2}{\left(I_{4}-1\right)}^{4}}\right)𝔹+\\ \left(8c_{0}c_{2}{\left(I_{4}-1\right)}^{3}{\text{e}}^{c_{1}{\left(I_{1}-3\right)}^{2}+c_{2}{\left(I_{4}-1\right)}^{4}}\right)\text{f}⊗\text{f}, \end{array} \end{array}$$

(15) $$\begin{array}{l} \sigma^{\text{W3}}=-p𝕀+2C_{1}𝔹+2a\left(I_{4}-1\right)e^{b{\left(I_{4}-1\right)}^{2}}\text{f}⊗\text{f}, \end{array}$$

in which 𝔹 = 𝔽𝔽^*T*^ is the left Cauchy–Green deformation tensor, and *p* is the Lagrangian multiplier to enforce the incompressibility constraint.

### 2.4. Experiments and Calibration

**Figure 3:**
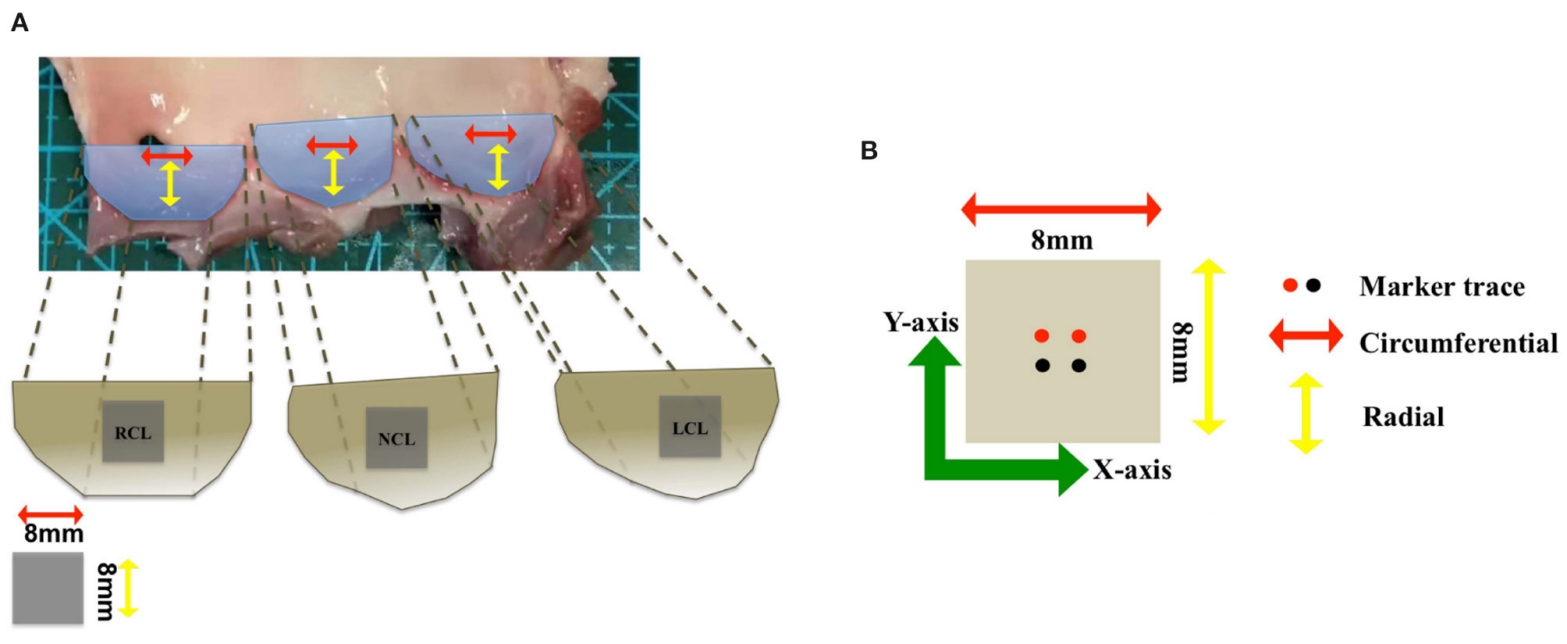
**(A)** The geometry of the aortic valve (AV) consists of three leaflets: right coronary leaflet (RCL), non-coronary leaflet (NCL), and left coronary leaflet (LCL). **(B)** The vector illustration shows the circumferential direction (red arrow) and radial direction (yellow arrow), and two different colors of the markers trace are used to mark the sample in two directions (X-axis) and (Y-axis).

In this section, we performed the tensile testing experiments using postmortem porcine AV samples from a domestic butcher house in Chongqing, China. The experimental protocols were similar to our previous study of *ex vivo* biomechanical tests on mitral valvular apparatus (Chen et al., 2020). In brief, squared samples were isolated from adult porcine hearts from the domestic butcher house (1-year old, ≥100 kg) and soaked in phosphate buffer saline (PBS) solution for moisture. The leaflet was cut into 8 × 8 mm samples on the middle part from the free edge and the edge of attachment to the aortic root (see Figure 3. Four square markers (1 × 1 mm) were glued to the surface with superglue (cyanoacrylate adhesive) for optical strain tracking as an illustration of the circumferential (X-axis) and the radial (Y-axis) directions. All samples were kept at 37 °C PBS bath and tested using a biaxial testing machine (BioTester) from CellScale to mimic the physiological loading condition. Eight preconditioning cycles were used to release the residual stress and adjust the tissue in a zero load. AV samples were then stretched to physiological stress estimated based on Laplace's law for a spherical surface assuming the mean radius of curvature of the AV to be 2 cm and the transvalvular pressure to be 120 mm Hg. The 1:1 stress ratio in two directions was applied to measure the anisotropic behaviors of the tissue. The displacements of markers and corresponding tensile forces were then recorded and calculated for stress and strain analysis. Details of the biaxial testing protocols can be found in Chen et al. (2020).

The parameters of the constitutive laws of the AV leaflets are obtained by minimizing the differences between the stretch–stress relationship derived from selected constitutive laws and corresponding experimental data. First, we obtained the experimental stretch–stress data from the biaxial testing. Second, the analytical stretch–stress relationships are obtained from Equations (13)–(15). Then, we perform the least square fitting following the same procedure in Cai et al. (2019) to determine the optimal parameters, and the *fmincon* function in Matlab is used to minimize the loss function, which is

(16) $$\begin{array}{l} f=\overset{n}{\underset{i=1}{\sum }}\left[{\left(\sigma_{11}^{\text{W}}-\sigma_{11}^{\text{exp}}\right)}^{2}+{\left(\sigma_{22}^{\text{W}}-\sigma_{22}^{\text{exp}}\right)}^{2}\right], \end{array}$$

where $\sigma_{11}^{\text{exp}}$ and $\sigma_{22}^{\text{exp}}$ are the experimental Cauchy stresses in the fiber and cross-fiber directions, and the superscript “W” indicates the stress is derived from a selected strain energy function.

In sum, we obtained nine sets of the biaxial stress tests from three porcine leaflets. Here, we further report the constitutive parameters by taking the average of nine sets of parameters, in which each set of parameters is obtained from one experimental sample. In the least square fitting, the lowest bounds for the iterative optimization parameters are set to be zero. The average R-squared value for each constitutive law is obtained by taking the average of nine sets of R-squared values, which is defined as R-squared = 1 − SSE/SST, where SSE is the residual sum of squares and SST is the total sum of squares. The closer the R-squared value to 1, the better the goodness-of-the-fitting.

### 2.5. The Numerical Implementation and Boundary Conditions

The whole AV model is immersed in an 8 × 8 × 14 cm fluid domain, which is further discretized into a regular 80 × 80 × 128 Cartesian grid. The time step size of 5e-6 s is selected because of the explicit time-stepping scheme. The detailed spatial and temporal discretizations can be found in Griffith and Luo (2017). The numerical implementation uses the IBAMR software infrastructure(https://github.com/IBAMR/IBAMR), which is a distributed-memory parallel implementation of the IB method with support for Cartesian grid adaptive mesh refinement. In this study, the Cartesian computational domain is discretized with 2 nested grid levels and a refinement ratio of 4 between the two levels. Note no refinement is applied to the structural mesh.

**Figure 4:**
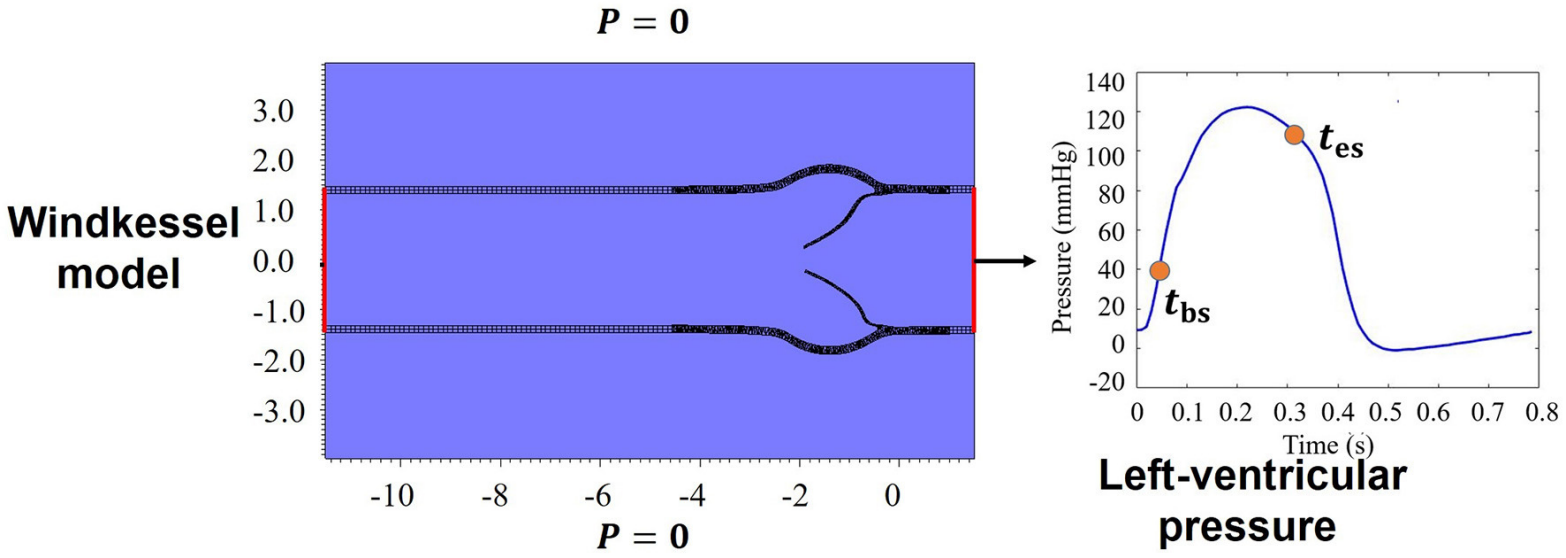
Boundary conditions for the aortic valve (AV) model.

A physiological LV pressure is used to drive blood flow through the AV, as shown in Figure 4. Meanwhile, a three-element Windkessel model is utilized to provide dynamic pressure loading of the aortic side for the AV model (Griffith, 2012), in which the outlet pressure of the Windkessel model is set as zero. The remaining boundaries of the FSI computational domain are with zero pressure, which is schematically illustrated in Figure 4. Furthermore, a large tethering force is applied at the outer surface of the aorta to keep the straight tube in place.

### 2.6. The Hemodynamic Parameters

To assess the hemodynamic performance of the AV, we introduce several hemodynamic parameters, including the regurgitant fraction, the mean transvalvular pressure gradient, the effective orifice area, and the energy loss of the LV. The regurgitant fraction reflects the regurgitant degree during valve closure and leakage. The regurgitant fraction (RF) is calculated by (Zhu et al., 2019).

(17) $$\begin{array}{l} \text{RF}=\frac{V_{R}+V_{L}}{V_{F}}\times 100\%, \end{array}$$

in which *V*_*F*_ is the forward volume, *V*_*R*_ is the regurgitant volume during the valve closing, and *V*_*L*_ is the leakage volume after the AV closes.

The mean transvalvular pressure gradient (TPG) measures the potential energy loss when the blood flows through the AV. The smaller the TPG, the smaller the potential energy loss (Yoganathan et al., 2004). The mean transvalvular pressure gradient during the systolic phase is calculated as

(18) $$\begin{array}{l} \text{TPG}=\frac{\int_{t_{\text{bs}}}^{t_{\text{es}}}\left(P_{\text{nlv}}-P_{\text{nao}}\right)dt}{t_{\text{es}}-t_{\text{bs}}}, \end{array}$$

in which *P*_nlv_ and *P*_nao_ are the pressures near the leaflets at the left ventricular and the aortic sides as shown in Figure 1, and *t*_bs_ and *t*_es_ are the beginning and end of systole as indicated in Figure 4.

To evaluate the impedance of the AV, the effective orifice area (EOA) is introduced as follows (Zhu et al., 2019):

(19) $$\begin{array}{l} \text{EOA}=\frac{Q_{\text{rms}}}{51.6\sqrt{\Delta P/\rho }}, \end{array}$$

(20) $$\begin{array}{l} Q_{\text{rms}}=\sqrt{\frac{\int_{t_{\text{bs}}}^{t_{\text{es}}}Q{\left(t\right)}^{2}dt}{t_{\text{es}}-t_{\text{bs}}}}, \end{array}$$

where *Q*(*t*) is the flow rate through the AV during the systolic phase at the center of the AV orifice as shown in Figure 1, *Q*_rms_ is the root mean square volumetric flow rate, and Δ*P* is the mean systolic transvalvular pressure gradient.

The energy loss of the LV (Zhu et al., 2017) is

(21) $$\begin{array}{l} E_{L}=0.1333\int_{t_{1}}^{t_{2}}\Delta p\left(t\right)Q\left(t\right)dt, \end{array}$$

where *t*_1_-*t*_2_ is the duration of one cardiac cycle, Δ*p* = *P*_Ao_ − *P*_LV_ is the aorta-left ventricular pressure difference, *P*_LV_ and *P*_Ao_ are the pressures at the center of the inlet and the outlet as shown in Figure 1, and *Q*(*t*) is the corresponding flow rate through the AV.

### 2.7. Summary of Simulated Cases

We simulate the AV dynamics with three different constitutive laws (W1, W2, and W3) and two different fiber architectures (FD1 and FD2), and all cases are denoted as W1FD1, W2FD1, W3FD1, W1FD2, W2FD2, and W3FD2. Cases W1FD1, W2FD1, and W3FD1 correspond to three different constitutive laws with the fiber architecture FD1, whereas cases W1FD2, W2FD2, and W3FD2 correspond to three different constitutive laws with the fiber architecture FD2. We perform the FSI simulations over two cardiac cycles to reach periodic convergence at the second period and onward, and one period lasts 0.8 s. Results are reported from the second period.

## 3. Results

### 3.1. The Experimental Fitting

The inferred constitutive parameters of three different constitutive laws (Equations 10–12) from the *ex vivo* porcine experiments are listed in Table 1.

**Table 1:** Fitted parameters for the three selected constitutive laws [Equations (10)–(12)].

	**Parameters**				**Average error (kPa)**	**Average R-squared**
	*C*_10_ (kPa)	*C*_01_	*k*_1_ (kPa)	*k*_2_		
W1	1.21	7.99	24.23	57.62	0.7 ± 0.63	0.99
	μ (kPa)	*c*_0_ (kPa)	*c*_1_	*c*_2_		
W2	1.18	55.04	8.08	54.00	0.75 ± 0.92	0.96
	*C*_1_ (kPa)	*a* (kPa)	*b*			
W3	19.59	12.94	77.79		1.72 ± 2.71	0.93

**Figure 5:**
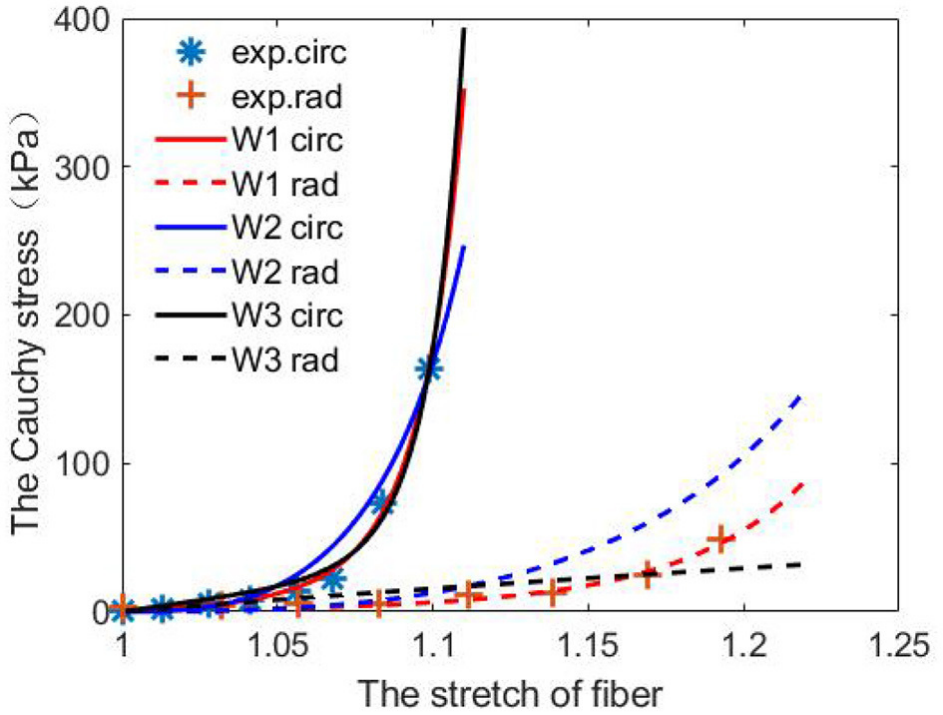
Predictions from the fitted three constitutive laws [Equations (10)–(12)] using one set of porcine experimental data.

From Table 1, we observe that the constitutive law W1 has the best agreement when fitting to the stretch–stress data from the porcine AV experiments, with the smallest error and highest R-squared score. While the constitutive law W3 is the poorest because of its incapability of describing the non-linear response along the cross-fiber direction. Figure 5 shows the fitted curves from the three constitutive laws compared to the porcine experimental data.

### 3.2. AV Opening and Closure

**Figure 6:**
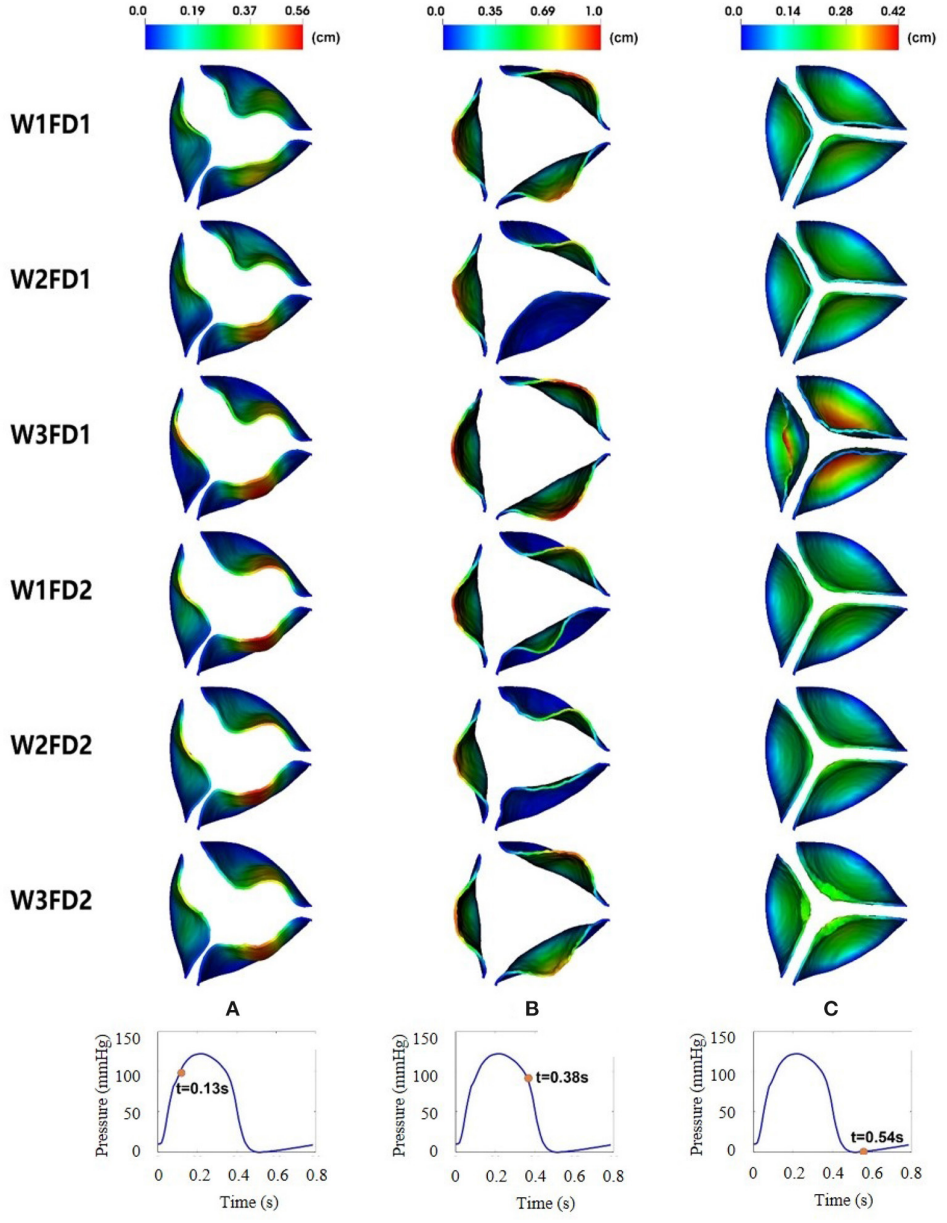
Dynamic deformation of the AV leaflets for the six cases at different time. The time points from left to right are 0.13 **(A)**, 0.38 **(B)**, and 0.54 s **(C)**.

Figure 6 shows the leaflets deformation of the AV from six cases within one cardiac cycle. At t = 0.13 s, the leaflet's deformation is similar for all six cases, especially the leaflet orifice area. At t = 0.38 s, the AV leaflets start to close and the closure inconsistency can be seen, especially in cases W2FD1, W1FD2, and W2FD2. Compared with other cases, the AV leaflets in case W2FD1 are the first to close, which may relate to the smallest orifice area of 1.19 cm^2^ at *t* = 0.38 s. At *t* = 0.54 s, the AV leaflets are at the fully closed state, with case W3FD1 of the largest displacements in the belly regions and the free edges of the leaflets.

Table 2 shows the average and maximum displacements of the AV from different cases at fully opened (0.18 s), pre-close (0.38 s), and fully closed (0.54 s) states during one cardiac cycle. When the AV is at the fully opened and pre-close states, the maximum displacements from case W3FD1 are slightly larger than those from other cases, reaching around 1.05 cm. This may be because case W3FD1 has the largest orifice area of 2.27 cm^2^ at the fully opened state and the largest orifice area of 2.01 cm^2^ at the pre-close state. Both cases W1FD1 and W1FD2 have larger maximum displacements compared with other cases when the AV is at the fully opened and pre-close states. At the pre-close state, the cases with FD1 have a larger maximum displacement than the cases with FD2 in general, which could be due to the larger orifice area of the cases with FD1. For example, the orifice area of case W1FD1 (1.92 cm^2^) is slightly larger than that of case W1FD2 (1.52 cm^2^). Compared with other constitutive laws, W2 has the smallest leaflets and displacements at the fully opened and pre-close states, which may suggest poor leaflet mobility at both opening and closing.

**Table 2:** Average and maximum displacements of AV with six different cases.

	**Average displacement (cm)**				**Maximum displacement (cm)**		
**Cases**	**Fully opened**	**Pre-close**	**Fully closed**		**Fully opened**	**Pre-close**	**Fully closed**
W1FD1	0.039	0.031	0.017		0.994	0.997	0.305
W2FD1	0.036	0.022	0.017		0.939	0.982	0.269
W3FD1	0.039	0.036	0.021		0.999	1.046	0.429
W1FD2	0.038	0.024	0.016		0.990	0.991	0.291
W2FD2	0.035	0.022	0.015		0.975	0.937	0.284
W3FD2	0.038	0.028	0.017		0.960	0.946	0.312

Table 3 shows the duration of different stages for the six cases. Here, the opening time is the duration from pre-open to fully opened states, the fully opened time is the duration from fully opened to pre-close states, and the closing time represents the duration from pre-close to fully closed states. The opening time for the six cases is similar, which is around 0.1 s. The fully opened state of six cases lasts about 0.14 s, with case W2FD1 having the shortest duration of 0.125 s. During the closure phase, the duration of the case W3FD1 reaches the longest (0.2 s), which could indicate poor leaflet mobility during closing (Zhu et al., 2019).

**Table 3:** The lasting time of different stages for the six cases (second).

	**W1FD1**	**W2FD1**	**W3FD1**	**W1FD2**	**W2FD2**	**W3FD2**
Opening	0.09	0.1	0.09	0.1	0.09	0.095
Fully opened	0.15	0.125	0.15	0.14	0.145	0.15
Closing	0.16	0.175	0.2	0.165	0.165	0.16

### 3.3. The Flow Pattern Comparison

**Figure 7:**
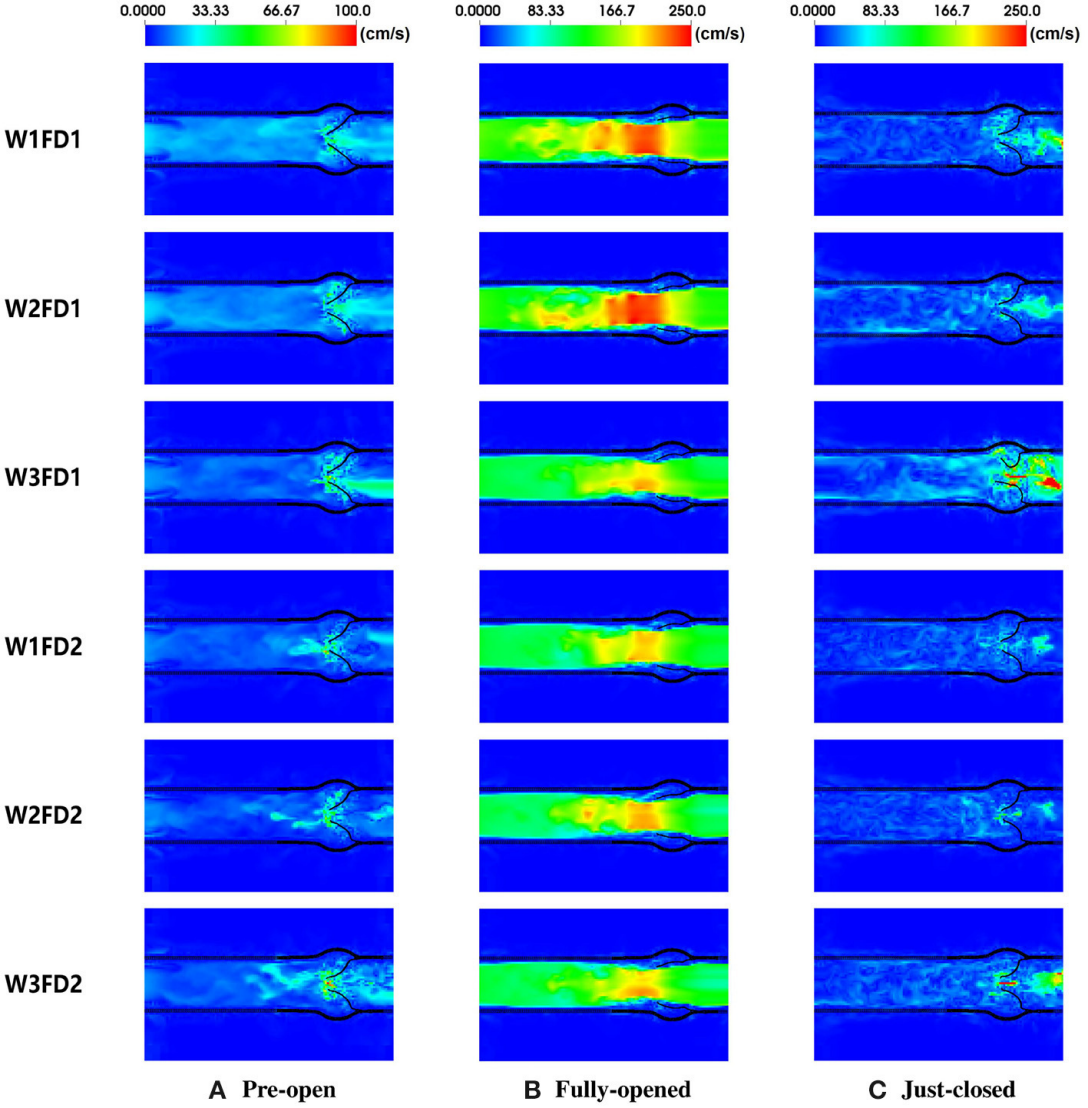
The fluid velocity field of the AV with six different cases. The time points from left to right are 0.09, 0.18, and 0.41 s, corresponding to **(A)** pre-open state, **(B)** fully opened state, and **(C)** just-closed state during the second cardiac cycle.

In this section, we compare the flow patterns of the AV from six cases. Figure 7 plots the fluid velocity field. When the AV starts to open, the blood gradually flows from the LV to the aorta. As the AV fully opens, there is a flow jet surging into the aorta. At the fully opened state, there exists a stronger jet flow in cases W1FD1 and W2FD1. At the just-closed state, there exists some regurgitant flow to facilitate the closure action, and case W3FD1 has the largest regurgitant flow. Comparing two different fiber architectures, the forward jet flow toward the aorta in cases with FD1 seems to be stronger than that of the cases with FD2. Similar results can be found for the regurgitant flow. On the other hand, cases with W1 and W2 have larger forward jet flow than the cases with W3, while W3 associates with the largest regurgitant flow.

Table 4 summarizes the peak blood velocity at the fully opened and just-closed states. When the AV fully opens, the peak velocities are from 2.0 to 3.0 m/s, consistent with the simulated flow velocity in Lee's study (Lee et al., 2020a). Compared with other cases, W1FD1 and W2FD1 have a larger peak velocity at the fully opened state. At the just-closed state, the peak regurgitation velocity of case W3FD1 reaches the largest 6.7 m/s. Except for the cases with W3, the peak forward velocity in cases with FD1 is slightly larger than that cases with FD2. Compared with other constitutive laws, cases with W3 experience the largest peak regurgitation velocity, which can be explained by the much longer closure duration.

**Table 4:** Peak velocity of transvalvular flow for the six cases.

	**Peak velocity of flow field (m/s)**	
**Cases**	**Fully opened**	**Just-closed**
W1FD1	2.47	2.47
W2FD1	2.57	2.00
W3FD1	2.17	6.74
W1FD2	2.14	2.20
W2FD2	2.26	3.52
W3FD2	2.21	3.65

**Figure 8:**
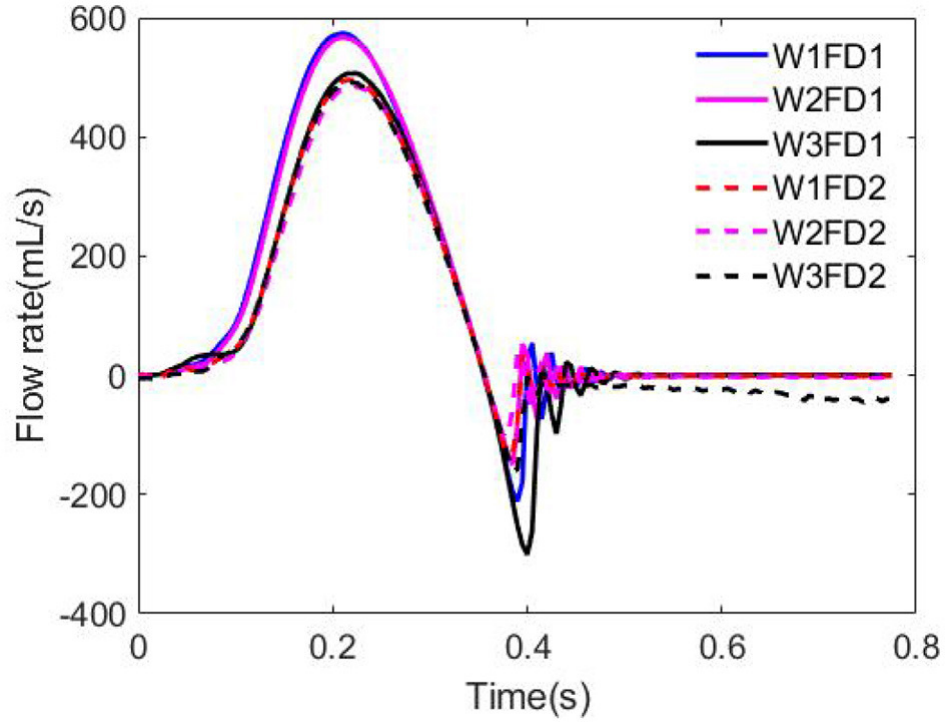
Flow rate comparison for the six cases.

Figure 8 shows the flow rates through the AV for the six different cases in one cardiac cycle. As the AV opens gradually, the blood flow ejects into the aorta driven by the fast increased LV pressure. Case W1FD1 experiences the largest peak flow rate of 574.05 mL/s and the largest forward volume 94.85 mL, which are slightly higher than the experimental data (Zhu et al., 2019; Lee et al., 2020a). Besides, the AV with FD1 generates a larger forward volume than that with FD2, for example, case W1FD1 can achieve 94.85 ml forward volume, higher than the value for W1FD2 (78.81 mL). During the closure, there exists a small regurgitant flow, with case W3FD1 generating the largest peak regurgitant flow rate 300.99 mL/s and the largest regurgitant volume 9.45 mL. Moreover, the regurgitant volume of the AV with FD1 is slightly larger than that with FD2, for example, the regurgitant volume of case W1FD1 (5.29 mL) is larger than case W1FD2 (3.09 mL). W1 has a slightly larger regurgitant volume than W2 in general. For example, the regurgitant volume of case W1FD1 (5.29 mL) is larger than case W2FD1 (3.07 mL). After the closure, case W3FD2 experiences the largest leakage volume 10.22 mL, which is beyond the reference value 2.81 mL. In summary, the cases with W1 have the largest peak forward flow rate and forward volume with a smaller peak regurgitant flow rate and a smaller regurgitant volume. While the cases with W3 have the largest peak flow rate of regurgitant flow and the largest regurgitant volume and the largest leakage volume. For the two different fiber architectures, the cases with FD1 have higher values in the peak forward flow rate and the forward volume than the cases with FD2. Furthermore, the oscillated flow rate after the AV closure is because of the FSI dynamics, which also appears in the MV simulations (Gao et al., 2014; Cai et al., 2019) and the AV simulations (Hasan et al., 2017; Lee et al., 2020a). In fact, the first peak regurgitate flow is the closure flow, which has been measured in the clinic (Hasan et al., 2017).

### 3.4. Strain and Stress Distributions

**Figure 9:**
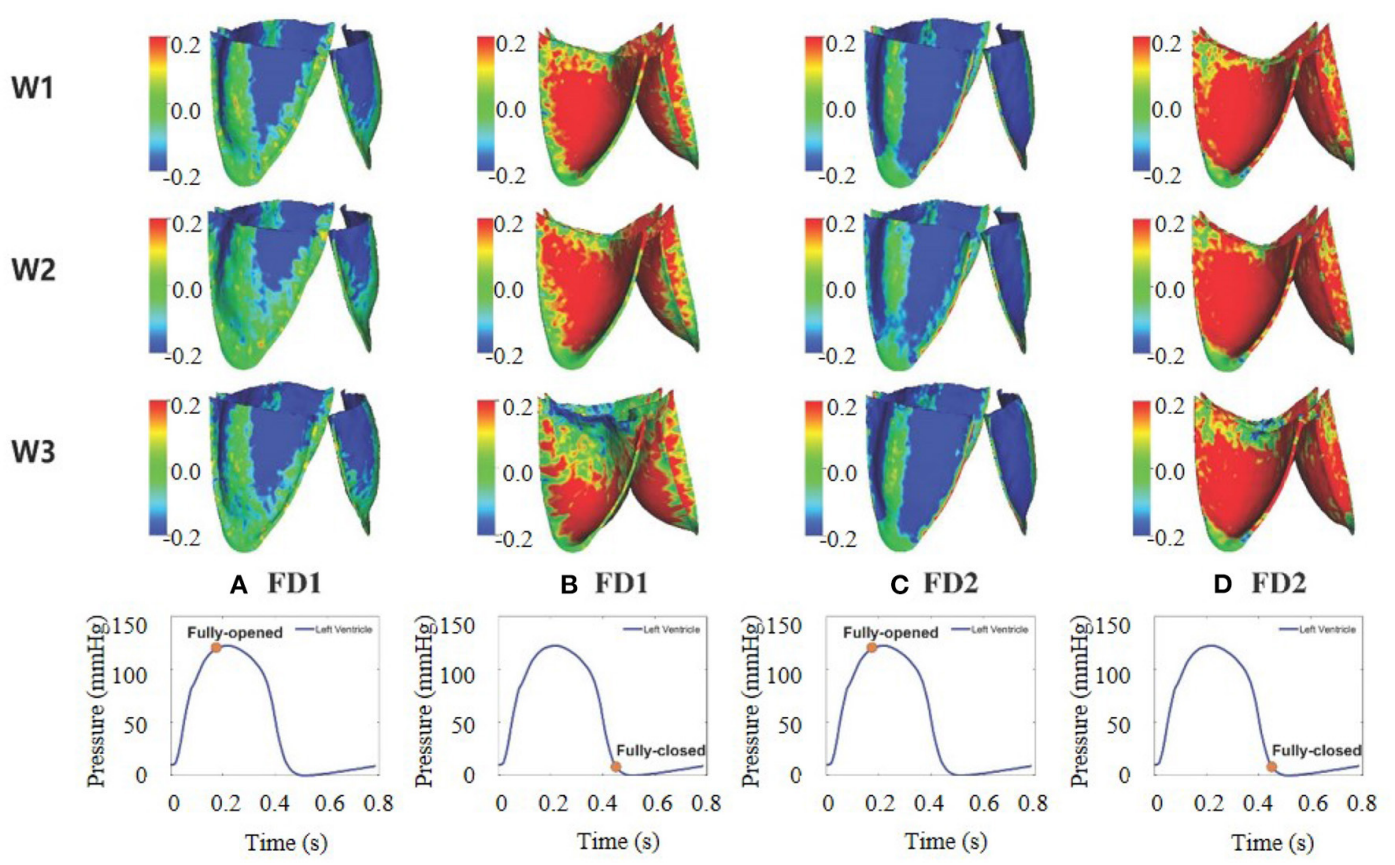
Fiber strain distribution of aortic valve (AV) leaflets for the six different cases. **(A–D)** The first two columns correspond to FD1 under the fully opened and fully closed states, and the last two columns correspond to FD2 under the fully opened and fully closed states.

**Figure 10:**
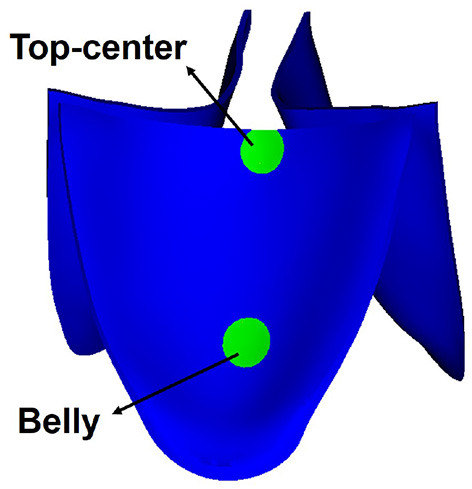
Two labeled regions of the aortic valve (AV) leaflet.

The fiber strain is defined as **f**_0_·(**E**·**f**_0_), in which $\text{E}=\frac{1}{2}\left({𝔽}^{T}𝔽-𝕀\right)$ is the Green strain tensor. The fiber stress is defined as **f**·(**σ** · **f**), in which **σ** is the Cauchy stress tensor. The fiber strain distributions for the six cases are shown in Figure 9. At the fully opened state, a large sporadic strain concentrates on the belly regions and the edges of the leaflets connected to the aortic wall. When the AV is at the fully closed state, the strain level of the entire leaflets reaches the largest, with case W3FD1 of the smallest strain distributional regions. Comparing two different fiber architectures, the AV with FD1 has a smaller compressed region than that with FD2 at the fully opened state. Besides, the AV with W1 shows a larger strain level of the belly region at the fully closed state. We further select two different regions of the AV leaflet, which are labeled as the belly region and the top-center region, as shown in Figure 10. Here, the belly region is defined as a circular region with the center at (−0.44, −0.64, −0.77) and a radius of 0.15 cm, and the top-center region with the center at (−0.19, −0.14, −1.76) and a radius of 0.15 cm. The average strain values of two different regions can be found in Table 5. When the leaflets are fully opened, all cases have negative strain values in the belly and top-center regions. When the leaflets are fully closed, the cases with W1 have the largest strain value in the belly and the top-center regions.

**Table 5:** Average fiber strain of aortic valve (AV) with six different cases.

	**Average fiber strain**				
	**Fully opened**			**Fully closed**	
**Cases**	**Belly**	**Top-center**		**Belly**	**Top-center**
W1FD1	−0.03	−0.06		0.32	0.10
W2FD1	−0.06	−0.06		0.25	0.07
W3FD1	−0.05	−0.11		0.27	−0.04
W1FD2	−0.13	−0.08		0.31	0.14
W2FD2	−0.14	−0.07		0.28	0.13
W3FD2	−0.10	−0.13		0.30	−0.01

The average stresses of the two selected regions are listed in Table 6. At the open state, the average stresses of the two regions are negative that is because of compression, in which the cases with FD2 have the larger compressed stress level than FD1 in the top-center region, while the cases with W2 have the largest compressed stress level in the belly region. Moreover, at the closed state, the cases with FD1 have a larger stress level in the belly region than that in the top-center region; on the contrary, the cases with FD2 experience a much larger stress level in the top-center region than that in the belly region. The previously published studies (Hasan et al., 2017; Lee et al., 2020a) have revealed that the fiber stress mainly concentrates on the belly region at the closed state, consistent with the stress distribution in the cases with FD1. Besides, the stress level of our AV is within a reasonable range compared with the reported stress levels in previous studies, for example, 150 kPa in Hasan et al. (2017) and the maximum stress level of the belly region 435 kPa in Sulejmani et al. (2019). Therefore, the stress distribution of FD1 seems to be consistent with the stress distribution of previous studies (Aggarwal et al., 2013; Hasan et al., 2017).

**Table 6:** Average fiber stress at the belly and top-center.

	**Average fiber stress (kPa)**				
	***t*** **= 0.32 s (open state)**			***t*** **= 0.47 s (closed state)**	
**Cases**	**Belly**	**Top-center**		**Belly**	**Top-center**
W1FD1	−36	−25		336	178
W2FD1	−41	−62		376	135
W3FD1	−22	−42		330	−4
W1FD2	−32	−48		309	480
W2FD2	−42	−92		295	555
W3FD2	−21	−43		262	54

### 3.5. Hemodynamic Parameter Comparison

Table 7 is the comparison of the hemodynamic parameters within the six cases. Here, we further present measurements from a tri-leaflet ePTFE valve (Zhu et al., 2019), from which this computational AV model is derived.

**Table 7:** The simulated hemodynamic parameters.

**Parameter**	**W1FD1**	**W2FD1**	**W3FD1**	**W1FD2**	**W2FD2**	**W3FD2**	**Reference AV (Zhu et al., 2019)**
RF (%)	6.73	4.47	13.67	5.22	4.90	18.16	14.37
TPG (mm Hg)	4.69	5.91	2.28	3.19	4.32	3.66	9.89
EOA (cm^2^)	3.41	3.00	4.25	3.51	2.95	3.25	2.86
E_L_ (mJ)	107.04	106.73	110.79	82.95	92.92	200.69	129.03

The least RF can be found for the cases using the constitutive law W2, while the cases with W3 have significantly higher RF, but interestingly closer to the measured values (14.37) (Zhu et al., 2019). The TPG values for all cases are much lower than the reference value 9.89 mm Hg, and the highest value is found in W2FD1 (5.91 mm Hg) with the lowest in W3FD1. Except for W3, the TPG values in cases with FD2 are lower than the values with FD1. Compared with other constitutive laws, the two cases of W2 have larger TPG in systole, suggesting a higher transvalvular potential energy loss with W2. The simulated EOA values are slightly higher than the reference value 2.86 cm^2^ from Zhu et al. (2019), the largest EOA is found in case W3FD1, suggesting the least resistance for blood flow. As for E_L_, the cases with FD1 have similar energy loss as the reported reference value (129.03 mJ), while E_L_ varies significantly for the cases with FD2 with the least energy loss for W1FD2 and the most energy loss for W3FD2.

### 3.6. Model Comparison and Selection

To select an appropriate constitutive law for the AV dynamics, we now propose a weighting scheme for the results from the *ex vivo* fitting, to the flow patterns, to the valvular strain/stress, and to the hemodynamic factor as shown in Table 8. For each selected criterion, we set the best value to be 1, and the worst value to be 0, respectively. Then we project each simulated result into the interval [0, 1]. By summing all values together for each case, we can rank the six cases from the most appropriate one to the poorest one. Specifically, the selected results and the corresponding criteria are as follows

- *Ex vivo fitting*: The least error suggests the best fitting of a constitutive law to experiments, we set the least error to be 1, and the largest error to be 0.
- *Opening orifice*: The opening orifice relates to the obstruction to blood flow, and the larger the opening orifice, the less obstruction to the blood flow. Thus, we consider the largest opening orifice area to be 1 and the smallest value to be 0.
- *Duration of AV opening*: As discussed in Zhu et al. (2019), the shorter the duration of AV opening, the better the leaflet mobility. In this aspect, we set the shortest duration of AV opening (0.09 s) to be 1, and the longest duration (0.1 s) to be 0.
- *Duration of AV closing*: Similar to the duration of AV opening, the shorter the duration of AV closing, the better the leaflet mobility. Thus, we set the shortest duration of AV closing (0.16 s) to be 1, and the longest duration of AV closing (0.2 s) to be 0.
- *The forward flow*: Generally, the large forward flow means a large stroke volume (Murgo et al., 1980). Thus, we set the largest forward volume to be 1, and the smallest value to be 0.
- *Regurgitation flow*: A small regurgitation flow will suggest the AV can close swiftly. To this end, we consider the smallest value to be 1, and the largest value to be 0.
- *Strain variation*: We consider a homogeneous strain distribution with small variation will be close to the physiological homeostasis (Kassab, 2008), and a smaller strain variation represents a higher degree of strain homogeneity. In this study, the strain variation is defined as the fiber strain difference between the belly and the top-center area at the fully closed state. For example, the strain variation of case W1FD1 can be calculated by 0.32 − 0.10 = 0.22. Then, we set 1 for the smallest strain variation and 0 for the largest strain variation.
- *Stress variation*: Similar to the strain variation, the stress variation is defined as the fiber stress difference between the belly and the top-center area at the closed state. Again, 1 for the smallest stress variation and 0 for the largest variation.
- *RF*: Similar to the *regurgitation volume*, we set 1 for the least value and 0 for the largest value.
- *TPG*: It relates to the flow potential energy losses when the blood flows through the AV (Yoganathan et al., 2004), thus we consider the smaller the TPG, the better performance of the AV dynamics.
- *EOA*: It is considered to be similar to the opening orifice.
- E_L_: The energy loss of the LV is also an important factor for assessing the AV performance (Zhu et al., 2019), in this aspect, we set the smallest E_L_ (82.95 mJ) to be 1.

Table 8 summarizes the weighting scores for the above 12 selected factors, with the total score in the last row. The rank for the six cases from the most appropriate one to the poorest one is

$$\begin{array}{l} \left\{\text{W1FD1},\;\text{W1FD2},\;\text{W2FD2},\;\text{W2FD1},\;\text{W3FD1},\;\text{W3FD2}\right\}. \end{array}$$

Case W1FD1 seems to be the best choice for modeling AV dynamics within the considered six cases, with non-linear anisotropic responses in the fiber and cross-fiber direction and a fully body-fitted fiber structure. The constitutive law W3 seems to perform poorest due to its linear response along the cross-fiber direction. In summary, the constitutive law W1 could be a good choice for modeling AV mechanical behaviors, and a fully body-fitted collagen fiber architecture is marginally better than using a simplified circumferentially aligned fiber architecture.

**Table 8:** Comparison of simulated results with scaled values.

	**W1FD1**	**W2FD1**	**W3FD1**	**W1FD2**	**W2FD2**	**W3FD2**
*Ex-vivo* fitting	1	0.95	0	1	0.95	0
Opening orifice	0.72	0	1	0.92	0.96	0.56
Duration of AV opening	1	0	1	0	1	0.5
Duration of AV closing	1	0.63	0	0.88	0.88	1
Forward flow	1	0.92	0.23	0.10	0	0.06
Regurgitation flow	0.56	0.86	0	0.86	1	0.74
Strain variation	0.56	0.81	0	0.88	1	0
Stress variation	1	0.53	0	0.93	0.42	0.72
RF	0.83	1	0.33	0.95	0.97	0
TPG	0.34	0	1	0.75	0.44	0.62
EOA	0.35	0.04	1	0.43	0	0.23
E_L_	0.8	0.8	0.76	1	0.92	0
Total	9.16	6.54	5.32	8.7	8.54	4.43

## 4. Discussion

In this study, we have used the IB/FE method to perform the FSI simulations of AV dynamics with three different constitutive laws and two different fiber architectures. The constitutive parameters of three different constitutive laws are first inferred from the experimentally measured stretch–stress data, which were from the *ex vivo* biaxial testing of three different porcine AV samples. By comparing the average errors and the average R-squared values, we observe that W1 is the most suitable constitutive law to describe the mechanical behaviors of those *ex vivo* AV leaflet samples. The simulation results also demonstrate that the constitutive law W1 has the larger leaflets displacements at the fully opened and pre-close states, the shorter duration for opening and closing, the largest peak forward flow rate, the largest forward volume, and the smaller regurgitant volume. The combination of the anisotropic non-linear constitutive law (W1) using exponential terms for both the fiber and cross-fiber directions and the fiber architecture with body-fitted orientation (FD1) has the shortest duration for AV opening and closing, the largest forward flow, the smallest stress variation, the less RF, and the smaller energy loss of the LV. Thus, our study seems to suggest that the constitutive law W1FD1 could be the most suitable model for simulating AV dynamics.

Figure 6 shows the dynamic deformation of the AV leaflets from six different cases. During the AV opening, the leaflet deformation is similar for the six cases, with a similar orifice shape at *t* = 0.13 s. Besides, the duration of AV opening is around 0.1 s, which is in good agreement with the value from Zhu's study (Zhu et al., 2019). During the AV closure, the closure inconsistency exists, which may relate to the AV model itself since it was reconstructed from the porcine pericardial valve. A similar phenomenon also appears in the bovine pericardial valve reported by Lee et al. (2020a). The duration of AV closure from the six cases is around 0.16 s, which is comparable to the value (0.14 s) reported by Zhu et al. (2019).

Figure 7 shows the fluid velocity fields of the AV. At the fully opened state, the peak velocity of the fluid field is within 2.0–3.0 m/s, which is in good agreement with the cross-sectional velocity fields reported in the previous studies (Flamini et al., 2016; Lee et al., 2020a). The peak forward flow rate is in a range from 480 to 580 mL/s, lower than the value 591.5 mL/s from Flamini's study (Flamini et al., 2016), but slightly higher than that of Lee's study (Lee et al., 2020a). The forward volume is within 75 to 95 mL, again slightly lower than the reported value 96.2 mL in Flamini's study (Flamini et al., 2016). At the just-closed state, the peak regurgitant velocity of the fluid field is slightly larger than the peak regurgitant velocity (less than 1.5 m/s) in Flamini et al. (2016); Lee et al. (2020a). The peak regurgitant flow rate is in reasonable agreement with the peak regurgitant flow rate (around 180 mL/s) compared to the value reported by Flamini et al. (2016).

Strain distributions of AV leaflets are shown in Figure 9. At the fully opened state, the AV leaflets experience some compression near the commissures, in which the AV with FD2 has more compressed regions than that with FD1. However, the compressed regions of the AV leaflets are much larger than those in Hasan's study (Hasan et al., 2017). While the strain distributions under the fully closed state are similar to those in Hasan's study (Hasan et al., 2017), especially in case W1FD1 and case W2FD1. For the stress distribution, the stresses are sporadically distributed on the belly regions and the edges connected with the aortic wall, different from Lee's study (Lee et al., 2020a). When the AV is at the fully closed state, the stresses are distributed symmetrically from commissure to commissure for both FD1 and FD2. At the fully closed state, the AV with FD1 experiences higher fiber stresses in the belly region of the leaflet, whereas the AV with FD2 (case W1FD2 and W2FD2) experiences higher fiber stresses in the top-center of the leaflet, which is caused by the large deformation of the top-center region.

Valvular morphology (three-leaflets or two-leaflets), size (aortic root diameter, leaflet area), geometrical shape (curvatures, thickness), and pathological state (calcification, etc.) can vary significantly among subjects, and those variations will have a significant impact on the AV dynamics and the corresponding flow patterns (Xiong et al., 2010; Zhu et al., 2020). The current study mainly focuses on the effects of different constitutive laws and different fiber architectures on the AV dynamic characteristics and the associated flow quantities, but not aims to simulate personalized AV dynamics. Therefore, an idealized healthy AV model is constructed based on the population-average anatomical measurements. A further limitation is the idealized aortic root. Studies (Flamini et al., 2016; Hasan et al., 2017) have shown that a personalized aorta will affect the blood flow, especially the flow jet across the AV, this would further affect the AV dynamics, such as the closure. For example, Flamini et al. (2016) have found that the aortic root can ensure a more efficient AV closure when using an elastic aortic root. Built on the same IB/FE framework, Hasan et al. (2017) studied the AV dynamics within an anatomically realistic aortic root and ascending aorta, and both were reconstructed from computed tomography angiography data; they found that their AV model can support a physiological diastolic pressure load without regurgitation, and it is able to accurately capture the leaflet biomechanics. Note that the present IB/FE approach can handle personalized AV models by simply replacing the idealized AV model, while it is challenging to reconstruct a personalized AV model from *in vivo* imaging data (Hasan et al., 2017), especially the collagen fiber structure.

In this study, a simplified 3-element Windkessel model is used for providing physiologically accurate pressure boundary conditions at the outlet. Although this 3-element Windkessel model has limitations to predict spatially distributed flow quantities, it is simple and accurate to predict the ventricular after-load as discussed by Westerhof et al. (2008). There are many blood flow models ranging from the zero-dimensional models (lumped-parameter models) (Liu et al., 2020), the one-dimensional models (Olufsen et al., 2000; Chen et al., 2016; Duanmu et al., 2019), and the three-dimensional models (Lee et al., 2016). Interested reader can refer to Shi et al. (2011); Morris et al. (2016) for reviews on blood flow modeling. Because of its simplicity, the lumped parameter models are still widely used to simulate the arterial hemodynamics (Westerhof et al., 2008; Fan et al., 2020). For example, Fan et al. (2020) constructed a closed-loop lumped parameter model including the LV, the systemic, and coronary circulations to describe the interactions between the LV and the coronary perfusion. As mentioned before, the lumped parameter model cannot assess the spatially distributed phenomena and wave propagation, being unable to capture the wave oscillations. To overcome those limitations, one-dimensional (1-D) models have been developed by taking into account geometrical measurements. For example, Chen et al. (2016) reported a coupled LV-systemic arteries model to study the effects of the arterial wall stiffness and vascular rarefaction on ventricular function. Using a similar 1-D arterial model, Duanmu et al. (2019) studied the coupling between the LV and the coronary blood flow. In this study, we do not intend to simulate patient-specific AV dynamics with detailed flow predictions in the systemic circulation, thus a 3-element Windkessel model is used. It is worth mentioning that this 3-element Windkessel model can be easily replaced by either other complex lumped parameter models or patient-specific 1-D/3-D blood flow models.

The blood flow around healthy valves is usually assumed to be laminar flow (Stijnen et al., 2004; Morbiducci et al., 2013; Wu et al., 2016; Pirola et al., 2018; Luraghi et al., 2019), while in the presence of diseased heart valves (obstructive and regurgitant valvular lesions) or prosthetic heart valves, the transition to turbulence exists (Stupak et al., 2017; Lee et al., 2020a). As suggested in Wei et al. (2018), individualized evaluations of turbulence may be needed. Turbulent models have been used in the simulations of blood flow around prosthetic AVs (Stupak et al., 2017), including direct numerical simulation (DNS), large eddy simulation (LES), and Reynolds-averaged Navier–Stokes (RANS). The turbulent model has not been employed in our AV simulations and other studies (Hasan et al., 2017; Lee et al., 2020a) using the same immersed boundary framework because explicit turbulent models have not yet been completely implemented in the present IB/FE approach. As discussed in Lee et al. (2020a), the current approach may be considered as an implicit large-eddy simulation with high-resolution slope limiters based on the piece-wise parabolic method. Because of the need for fine temporal and spatial discretization, and tremendous computational cost to capture small-scale turbulent flow features in the present approach, therefore, local flow features are not reported in the present study, but more on the average flow quantities, such as flow rate, pressure, and so on. Further limitations include (1) the biaxial tests were conducted in porcine AV samples, but not from human AV leaflets; (2) personalized human AV model is not used in this study; and (3) a typical LV pressure profile is used, but not from a realistic human heart model Chen et al. (2016); Gao et al. (2017a).

## 5. Conclusion

In this study, we construct an idealized AV model coupled with a three-element Windkessel model. Three different anisotropic hyperelastic material models and two different symmetric fiber architectures are used for modeling the AV leaflet mechanics. By using the IB/FE method, FSI simulations of six different cases are performed in this study. Our results are in reasonable agreement with the previous experimental and numerical studies of AV dynamics, especially the hemodynamic performance. Finally, the comparison shows that the combination of an anisotropic non-linear constitutive law using exponential terms for both the fiber and cross-fiber directions and the fiber architecture with body-fitted orientation could be suitable for characterizing the AV dynamics and its hemodynamic performance. Although there exist some limitations, our results provide references for selecting a proper material model and fiber architecture for FSI modeling of the AV dynamics.

## Data Availability Statement

The original contributions presented in the study are included in the article/Supplementary Material, further inquiries can be directed to the corresponding author/s.

## Ethics Statement

Ethical review and approval was not required for the animal study because we performed the tensile testing experiments on post-mortem porcine AV samples from a domestic butcher house in Chongqing, thus ethical approval is not needed.

## Author Contributions

RZ and LC performed numerical modeling and wrote the manuscript. YL and GZ provided the computational AV model. XM conducted the *ex-vivo* experiments. YW assisted result analysis. HG critically reviewed the manuscript. LC, XL, and HG supervised the overall project. All authors analyzed the results, read, and edited the manuscript.

## Conflict of Interest

The authors declare that the research was conducted in the absence of any commercial or financial relationships that could be construed as a potential conflict of interest.
