# Supplementary material for: The Comparison of Different Constitutive Laws and Fiber Architectures for the Aortic Valve on Fluid–Structure Interaction Simulation
Source: Front Physiol. 2021 Jun 24;12:682893. doi: 10.3389/fphys.2021.682893 (PMC8266211; doi:10.3389/fphys.2021.682893)
Supplement: Supplementary file 1 [file Data_Sheet_1.PDF]

Supplementary Material

1 THE EXPERIMENTAL DATA

The initial experimental stretch-stress data is listed in Table S1 for the first porcine AV sample, Table S2 for the second porcine AV sample, and Table S3 for the third porcine AV sample. In each table, LCL, NCL, and RCL represent the experimental stretch-stress data of the left coronary leaflet, non-coronary leaflet, and right coronary leaflet, respectively. Here, the stress refers to the first Piola-Kirchhoff stress.

Table S1. Initial experimental stretch-stress data of the first AV sample.

0401LCL			
X STRETCH	X PK1(MPa)	Y STRETCH	Y PK1(MPa)
1	-0.000392411	1	0.005694923
1.019976863	0.000585537	1.020763296	0.005678479
1.041666667	0.001556789	1.045652676	0.005683049
1.062777766	0.001551217	1.070243363	0.005683714
1.085370382	0.008128593	1.097909303	0.006653996
1.105057893	0.013870657	1.12198011	0.007604003
1.123263889	0.024330487	1.147411515	0.012365988
1.146423622	0.051682091	1.173418163	0.019057659
1.160011574	0.106852286	1.194712411	0.029462464
0401NCL			
X STRETCH	X PK1(MPa)	Y STRETCH	Y PK1(MPa)
1	0.002398024	1	0.001938172
1.016741233	0.00237901	1.01796083	0.00386947
1.037376965	0.002370253	1.041183847	0.006794339
1.058171385	0.007085416	1.06370544	0.006808586
1.076509643	0.011744083	1.086697268	0.007802669
1.093569604	0.020762134	1.114005411	0.007852574
1.109952429	0.056301599	1.141298513	0.014813594
1.123009123	0.130924323	1.165490536	0.026951653
1.125320894	0.139273812	1.179862193	0.028782656
1.127019881	0.13977574	1.188467538	0.032411775
1.126987692	0.140138026	1.195649359	0.038816022
1.127695592	0.140619117	1.203315614	0.045123858
1.129353428	0.142488953	1.209531805	0.058991406
0401RCL			
X STRETCH	X PK1(MPa)	Y STRETCH	Y PK1(MPa)
1	0.00107145	1	0.003189223
1.01317044	0.002375212	1.031941515	0.00429237
1.028155721	0.004954213	1.056529268	0.005375995
1.040908625	0.007455482	1.082460119	0.005392779
1.055906901	0.013729785	1.111715439	0.010852018
1.067648602	0.020816264	1.138324494	0.010890515
1.084010983	0.067596606	1.16889629	0.020832892
1.099058902	0.149274257	1.192845771	0.040833741

Table S2. Initial experimental stretch-stress data of the second AV sample.

X STRETCH	X PK1(MPa)	Y STRETCH	Y PK1(MPa)
0402LCL			
X STRETCH	X PK1(MPa)	Y STRETCH	Y PK1(MPa)
1	0.000399604	1	0.003214263
1.019785509	0.004368325	1.041193182	0.002089137
1.037821591	0.01399326	1.08147731	0.006795358
1.050496635	0.033279326	1.119829582	0.006842213
1.066378232	0.076988642	1.159583352	0.015219647
1.070277995	0.113108798	1.199829564	0.02492641
0402NCL			
X STRETCH	X PK1(MPa)	Y STRETCH	Y PK1(MPa)
1	0.000479389	1	0.002757691
1.012677145	0.005832032	1.028085443	0.003668655
1.027576901	0.020582516	1.060126582	0.011023719
1.04601908	0.06178162	1.086250015	0.024878049
1.053639538	0.093219636	1.108813322	0.046517282
0402RCL			
X STRETCH	X PK1(MPa)	Y STRETCH	Y PK1(MPa)
1	-0.001036918	1	0.002311178
1.021710526	0.001034077	1.017824521	0.003108274
1.048657869	0.003998869	1.03638215	0.00389423
1.073342092	0.012887292	1.054525086	0.005471615
1.092078922	0.031096509	1.065572997	0.010155115
1.107236842	0.064928941	1.071973129	0.016201859
1.116447368	0.08790035	1.091309777	0.024784294

2 FITTING CODE

Take the constitutive law W1 as an example, the corresponding fitting procedures are as follows. Here, **avfitconstraint.m** denotes the objective function to be optimized, while **avconstraintfigure.m** is used to iteratively obtain parameters and plot the fitting curve.

avfitconstraint.m

```
function f=avfitconstraint(cf)
A=xlsread('AOV.xlsx');
lam1=A(3:11,1)';
lam2=A(3:11,3)';
p1=A(3:11,2)';
p2=A(3:11,4)';
sigma1=lam1.*p1;
sigma2=lam2.*p2;
f=0.0;
for i=1:9
f=f+(2*cf(1)*cf(2)*exp(cf(2)*(lam1(i)^2+lam2(i)^2+1./(
    lam1(i)^2*lam2(i)^2)-3))*(lam1(i)^2-1./(lam1(i)^2*
    lam2(i)^2))+2*cf(3)*(lam1(i)^2)*(lam1(i)^2-1)*exp(cf
    (4)*((lam1(i)^2-1)^2))-sigma1(i))^2;
```

Table S3. Initial experimental stretch-stress data of the third AV sample.

0403LCL			
X STRETCH	X PK1(MPa)	Y STRETCH	Y PK1(MPa)
1	0.000498776	1	0.00211568
1.012809535	0.001723694	1.012241861	0.005241344
1.023809524	0.002913848	1.030570652	0.006268339
1.037785703	0.011049405	1.046209252	0.00830782
1.05	0.034654405	1.059809809	0.010308471
1.070833333	0.105973139	1.07202447	0.014313071
1.073214286	0.146383346	1.088328818	0.017298189
0403NCL			
X STRETCH	X PK1(MPa)	Y STRETCH	Y PK1(MPa)
1	0.001167624	1	0.00493647
1.021403414	0.001163878	1.012987298	0.004890508
1.045373618	0.002327224	1.028601695	0.004858072
1.068764427	0.00697374	1.04476696	0.005879465
1.090167841	0.016230787	1.060932224	0.007935482
1.111023947	0.047154335	1.078411038	0.006863208
1.12812853	0.135568436	1.092711885	0.013010933
1.129877414	0.14035649	1.112298739	0.011954144
0403RCL			
X STRETCH	X PK1(MPa)	Y STRETCH	Y PK1(MPa)
1	0.001710198	1	0.001300072
1.020887944	0.003427246	1.034650989	0.006171875
1.043160316	0.004298653	1.071244469	0.00943757
1.065600563	0.007763004	1.105260708	0.007797361
1.089421601	0.012995709	1.130844416	0.009339815
1.112532556	0.015479613	1.153508654	0.012422282
1.132783403	0.034475527	1.175502687	0.028350642
1.146795967	0.054962649	1.199478783	0.051539164
1.158136031	0.090628747	1.221172947	0.103917241

```
end
for j=1:9
f=f+(2*cf(1)*cf(2)*exp(cf(2)*(lam1(j)^2+lam2(j)^2+1./(lam1(j)^2*lam2(j)^2)-3))*(lam2(j)^2-1./(lam1(j)^2*lam2(j)^2))-sigma2(j))^2;
end
```

avconstraintfigure.m

```
clc
clear
format long
```

```
A=xlsread('AOV.xlsx');
lam1=A(3:11,1)';
lam2=A(3:11,3)';
p1=A(3:11,2)';
p2=A(3:11,4)';
sigma1=lam1.*p1;
sigma2=lam2.*p2;
plot(lam1,sigma1,'*',lam2,sigma2,'+');
hold on
N=100;
dl1=(max(lam1)-min(lam1))/N;
dl2=(max(lam2)-min(lam2))/N;
for i=1:N
lam11(i)=lam1(1)+i*dl1;
lam22(i)=lam2(1)+i*dl2;
end
cf0=[3.02e-4 3.25 0.197 0.001];
lb=[0 0 0.1 0];
ub=[inf inf inf inf];
[cf,fval]=fmincon('avfitconstraint',cf0,[],[],[],[],lb,
    ub,[])
for i=1:N
sigma1f(i)=2*cf(1)*cf(2)*exp(cf(2)*(lam11(i)^2+lam22(i)
    ^2+1./(lam11(i)^2*lam22(i)^2)-3))*(lam11(i)^2-1./(
    lam11(i)^2*lam22(i)^2))+2*cf(3)*(lam11(i)^2)*(lam11(i
    )^2-1)*exp(cf(4)*((lam11(i)^2-1)^2));
end
for j=1:N
sigma2f(j)=2*cf(1)*cf(2)*exp(cf(2)*(lam11(j)^2+lam22(j)
    ^2+1./(lam11(j)^2*lam22(j)^2)-3))*(lam22(j)^2-1./(
    lam11(j)^2*lam22(j)^2));
end
plot(lam11,sigma1f,'r-',lam22,sigma2f,'b-')
xlabel('The stretch of fiber');
ylabel('The Cauchy stress (MPa)');
hold on
```

3 ALL FITTING CURVES

The nine sets of fitting curves are shown in figure S1, in which each subfigure includes the fitting curves of three different constitutive laws to our porcine experimental data. Here, the left column, middle column, and right column represent the fitting for the experimental stretch-stress data of LCL, NCL, and RCL, respectively. By comparison, the constitutive law W1 has the best fitting effect for our porcine experimental

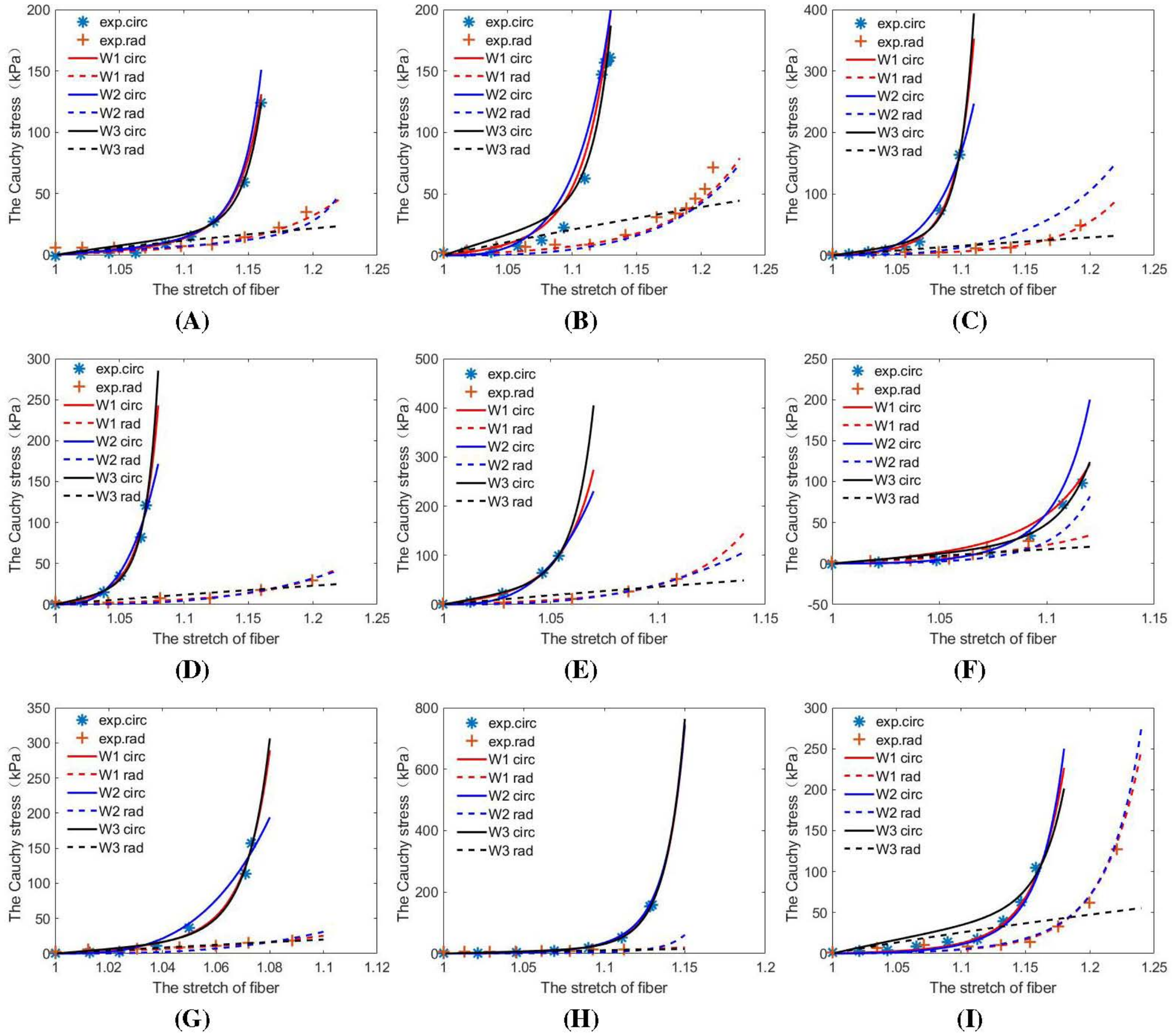

Figure S1. Fitting three constitutive laws to our biaxial porcine experimental data: The first porcine AV sample (panels (**A**), (**B**) and (**C**)), second porcine AV sample (panels (**D**), (**E**) and (**F**)), third porcine AV sample (panels (**G**), (**H**) and (**I**)).

data, then W2, finally W3.
